# [Corrigendum] Circular RNA expression profiles significantly altered in UVA-irradiated human dermal fibroblasts

**DOI:** 10.3892/etm.2026.13280

**Published:** 2026-09-01

**Authors:** Mengbi Lin, Yue Zheng, Qian Li, Yufang Liu, Qingfang Xu, Yuying Li, Wei Lai

Exp Ther Med 20:153, 2020; DOI: 10.3892/etm.2020.9292

Following the publication of this paper, it was drawn to the authors' attention by a concerned reader that one of the antibodies the authors used in their study was apparently selected inappropriately: The antibody (cat. no. ab51243) that the authors reported to have used for the apoptosis-associated protein p16-INK4a was in fact specific for the protein known as P16 ARC (or ARPC5), not as the authors had intended.

The authors replied to explain that they had made a typing error in their paper. They confirmed that they did use the correct antibody for the immunostaining experiments {cat. no. ab108349; anti-CDKN2A/p16INK4a antibody [EPR1473]}, rather than antibody cat. no. ab51243, and they were also able to show to the Editorial Office the documents providing proof of the purchase of the antibody.

Therefore, in the “*Detection of cell cycle-associated protein expression*” subsection of the Materials and methods section on p. 2, left-hand column, the relevant text on line 10 should have read as follows (the changed text is highlighted in **bold**): “...rabbit P16 (1:1,000 dilution; cat. no. **ab108349**; Abcam).

The authors are grateful to have been given the opportunity to clarify this issue in their paper. All the authors agree to the publication of this Corrigendum, and are grateful to the Editor of *Experimental and Therapeutic Medicine* for granting them the opportunity to publish this; moreover, they thank the interested reader for drawing this matter to their attention, and apologize to the readership for any inconvenience caused.

